# Pharmacophore-Model-Based Virtual-Screening Approaches Identified Novel Natural Molecular Candidates for Treating Human Neuroblastoma

**DOI:** 10.3390/cimb44100329

**Published:** 2022-10-13

**Authors:** F A Dain Md Opo, Saleh Alkarim, Ghadeer I. Alrefaei, Mohammad Habibur Rahman Molla, Nouf H. Alsubhi, Faisal Alzahrani, Foysal Ahammad

**Affiliations:** 1 Department of Biological Science, Faculty of Sciences, King Abdulaziz University (KAU), Jeddah 21589, Saudi Arabia; 2 Embryonic Stem Cell Research Unit, King Fahd Medical Research Center (KFMRC), King Abdulaziz University (KAU), Jeddah 21589, Saudi Arabia; 3 Embryonic and Cancer Stem Cell Research Group, King Fahd Medical Research Center (KFMRC), King Abdulaziz University (KAU), Jeddah 21589, Saudi Arabia; 4 Department of Biology, College of Science, University of Jeddah, Jeddah 21589, Saudi Arabia; 5 Department of Biological Sciences, College of Science & Arts, King Abdulaziz University (KAU), Rabigh 21911, Saudi Arabia; 6 Division of Biological and Biomedical Sciences (BBS), College of Health and Life Sciences (CHLS), Hamad Bin Khalifa University (HBKU), Doha 122104, Qatar

**Keywords:** neuroblastoma, *MYCN*, Brd4, molecular docking, pharmacophore model, side effects, dynamic simulation

## Abstract

The mortality of cancer patients with neuroblastoma is increasing due to the limited availability of specific treatment options. Few drug candidates for combating neuroblastoma have been developed, and identifying novel therapeutic candidates against the disease is an urgent issue. It has been found that *muc-N* protein is amplified in one-third of human neuroblastomas and expressed as an attractive drug target against the disease. The *myc-N* protein interferes with the bromodomain and extraterminal (BET) family proteins. Pharmacologically inhibition of the protein potently depletes *MYCN* in neuroblastoma cells. BET inhibitors target *MYCN* transcription and show therapeutic efficacy against neuroblastoma. Therefore, the study aimed to identify potential inhibitors against the BET family protein, specifically Brd4 (brodamine-containing protein 4), to hinder the activity of neuroblastoma cells. To identify effective molecular candidates against the disease, a structure-based pharmacophore model was created for the binding site of the Brd4 protein. The pharmacophore model generated from the protein Brd4 was validated to screen potential natural active compounds. The compounds identified through the pharmacophore-model-based virtual-screening process were further screened through molecular docking, ADME (absorption, distribution, metabolism, and excretion), toxicity, and molecular dynamics (MD) simulation approach. The pharmacophore-model-based screening process initially identified 136 compounds, further evaluated based on molecular docking, ADME analysis, and toxicity approaches, identifying four compounds with good binding affinity and lower side effects. The stability of the selected compounds was also confirmed by dynamic simulation and molecular mechanics with generalized Born and surface area solvation (MM-GBSA) methods. Finally, the study identified four natural lead compounds, ZINC2509501, ZINC2566088, ZINC1615112, and ZINC4104882, that will potentially inhibit the activity of the desired protein and help to fight against neuroblastoma and related diseases. However, further evaluations through in vitro and in vivo assays are suggested to identify their efficacy against the desired protein and disease.

## 1. Introduction

Human cells carry cell cycle-based active enhancers, promoter regions, and a number of super-enhancers. Through analysis of myeloma cell lines, 10,000 active transcription sites, about 8000 enhancers, and 308 super-enhancers have been identified [1]. The bromodomain and extraterminal domain (BET) protein, which maintains gene expression, is one of the epigenetic readers [2]. BET proteins operate as substrates to draw several other proteins to the promoter region and at the enhancer of active genes, particularly super-enhancer genes, and promote transcription by recruiting kinetics as well as interdependency [1,3]. BET series of proteins (Brd2, Brd3, Brd4, and Brdt) are chromatin reader proteins well known for regulating the transcription of genes that participate in oncogenesis and cell proliferation [4]. Different hematologic cancer models show that Bet protein antagonists can produce an anti-proliferative effect partly because they repress the *MYCN* oncogene by inhibiting the Myc-driven pathway [5]. The MYC family of oncogenes includes *n-myc*, *c-myc* (MYC), and *l-myc* and usually expresses in the central nervous system, usually in the forebrain and hindbrain [6,7]. The structure of the myc-N protein comprises 464 amino acid (AA) residues along with two domains, namely N and C. The N-terminus domain confers the ability to activate the transcription of genes associated with the production of the myc-N family protein, whereas the C-terminal conserved domain is responsible for DNA binding [6]. Overexpression of the *MYCN* gene causes cancer, specifically neuroblastoma, T-cell leukemia, glioblastoma, and breast adenocarcinoma [8,9]. Through the recruitment and maintenance of the pTEFb complex at gene promoters during mitosis, BRD4 controls various genes vital for cell function. *MYCN* suppression was varied in a neuroblastoma cell line that expresses myc-N, so the regulation of MYC and the cellular response to BET inhibition differ between hematologic and solid tumor types, such as neuroblastoma [10,11]. BET antagonist’s able to block myc-N over expression might occasionally be linked to their anti-cancer effect, although in some cases, these effects are controlled by a distinct group of cancer-related genes [12]. To date, no specific therapeutics have been developed targeting the protein that can be used to fight against the diseases.

Neuroblastoma is the most predominant cancer among children, and the two forms of the disease include localized and metastatic [13]. The survival rate is lower for metastatic tumors, typically less than 40%. Early-stage detection and diagnosis of the tumor play a vital role in the overall survival rate of cancer. If diagnosed early, at less than one year, the tumors can often be easily cured. However, diagnoses after one year have an increased possibility of metastases, with a low survival rate, and require rigorous chemotherapy and other treatment option [14,15]. In most cases, patients resistant to therapies have been observed to have Brd4 amplification along with *MYCN* [16]. Targeting Brd4 protein is essential in treating several cancers, and few chemical synthesis drugs have been developed that can reduce its overexpression by inhibiting transcription and direct target-based approaches [9]. Retinoic acid works at the mRNA level to downregulate protein expression via cell cycle arrest in neuroblastoma treatment [17]. It has been observed in-vivo studies that several inhibitors can target BET protein in case of acute myeloid leukemia (AML), myeloma, and lymphoma. Proto-oncogenes like Myc, as well as b-cell lymphoma 2 (BCL 2), can suppress transcriptional blood cancer cells and neuroblastoma through the exposure to the BET inhibitors [18]. BET bromodomain inhibitors lead to the differentiation of the T cells, which leads to producing therapeutic effects in autoimmune disease and also suppresses the overexpression of the *MYC* through transcriptional effects in treating leukemia cells [5,19].

Nowadays, using chemically synthesized compounds as treatment for neuroblastoma has shown long-term side effects, including growth impairment, neurological disorders, the development of leukemia, and hormonal imbalances [20]. Using natural compounds instead of chemically synthesized compounds may help to reduce the side effects arising from the treatment of previously developed drugs.

Computer-aided drug design (CADD), also known as in silico drug design, is currently a popular approach to designing drugs against a specific target. Modern CADD approaches, including pharmacophore modeling, molecular docking, ADMET (absorption, distribution, metabolism, excretion, and toxicity), molecular dynamics (MD) simulation, and MM-GBSA (Molecular mechanics with generalized Born and surface area solvation), can generate hits to lead compounds more rapidly compared to the conventional drug design process from the ZINC database through virtual screening as it contains several libraries of well-characterized [21,22,23]. For example, molecular docking can initially help determine the binding affinity of the ligands with the target protein that shows a desired biological response measure through in vitro and in vivo methods [24]. In conventional drug development, the ADMET parameters can be evaluated by obtaining blood samples from the clinical trial stage or in vivo [25,26]. However, CADD approaches utilize different techniques that can evaluate the ADMET parameters more easily and efficiently. The techniques can determine a broad range of organ and other toxicity properties, indicating the possibility of contradictory effects [27]. Where the MD simulation can determine the stability of the compound to the target protein in different time scales, MM-GBSA can subsequently determine the binding free energy of a drug-like compound to the target protein [16]. CADD approaches facilities to identify drug-like compounds more easily. Therefore, this study aimed to identify potential natural compounds against neuroblastoma disease using pharmacophore modeling, virtual screening, molecular docking, ADMET, MD simulation, and MM-GBSA approaches. The natural lead compounds identified through the modern CADD approaches will be able to reduce the overexpression of the BET protein and, subsequently, the overexpression of *MYCN* proto-oncogene, resulting in hindering the activity of the neuroblastoma and related disease.

## 2. Results

### 2.1. Development of Pharmacophore Model

**Figure 1:**
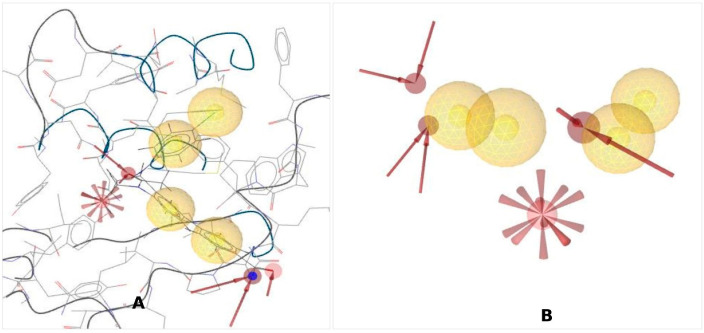
Representing a structure-based pharmacophore model generated from the PDB ID: 4BJX in complex with the attached ligand (PDB ID: 73B). (**A**) Generated features and protein structure (**B**) pharmacophore features without protein chain. There are four hydrophobic interactions (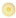), red arrows (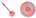) indicating H-bond acceptors, and one red star (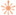) depicting negative ionizable groups.

To identify novel scaffolds as a lead compound against Brd4 protein, structure-based pharmacophore models were generated based on the protein data bank (PDB) ID: 4BJX in complex with the ligand PDB ID: 73B. The inhibitory activity of the ligand 73B with the Brd4 protein has confirmed previously through experimentally inhibitory concentration (IC_50_): 21 nM, and validated by X-ray diffraction, resolution: 1.59 Å, R-value free: 0.185, indicating that this series of inhibitors could bind the active site of the protein subsequently inhibition of their biological activity. Therefore, the Brd4 protein (4BJX) structure was used to generate the pharmacophore model in this study, and Ligand Scout 4.4 Advance molecular design software was used to create the pharmacophore model (Figure 1A). Analysis of the complex 3D structure of the protein found six hydrophobic contacts, two hydrophilic interactions, one negative ionizable bond, and fifteen exclusion volumes presented in Figure 1B.

### 2.2. Validation of Pharmacophore Model

Validation is a crucial parameter for an authentic pharmacophore model, as well as determining the model’s quality. To validate the pharmacophore model initially, 36 active antagonists of the selected protein were identified from a literature search and the online ChEMBL database (Table S1). Subsequently, the active compounds were submitted to the DUD-E decoy database, and corresponding decoy compounds were retrieved.

**Figure 2:**
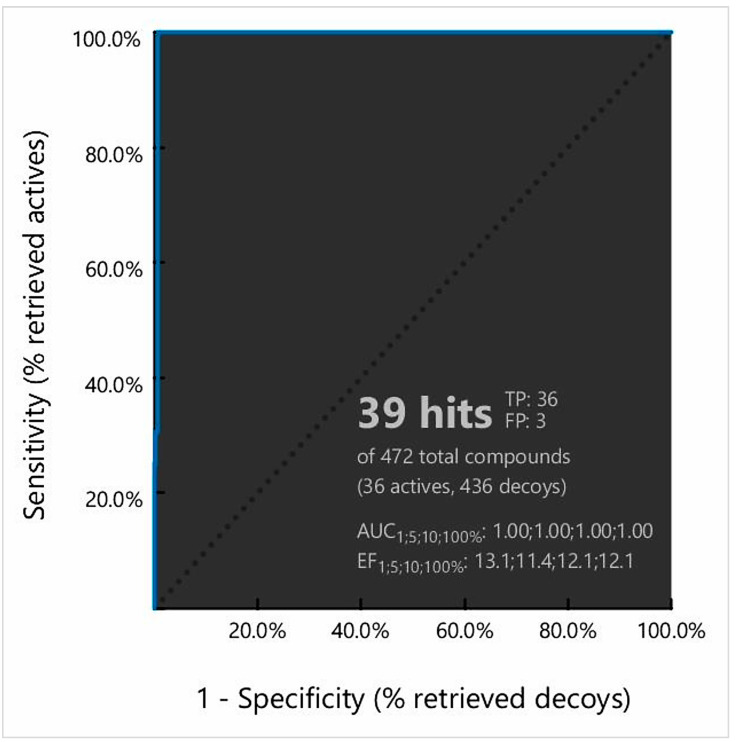
Representing a receiver operating characteristic (ROC) curve obtained during the pharmacophore model validation process. The total number of active and decoy sets was determined using the ChEMBL and DUD-E decoy databases, respectively.

The active compounds and corresponding decoy sets were used to validate the pharmacophore model. Initially, the pharmacophore model-based screening process generated a ROC curve based on the discrimination ability of the model from active to inactive. The obtained sensitivity curve (ROC curve) indicated both specificity and sensitivity. The quality of the curve was shown by the area under the curve (AUC) and the enrichment factor (EF) value. AUC values ranging from 0 to 0.5 suggest a likelihood of discrimination; acceptable ranges were 0.51 to 0.7 and 0.71 to 0.8, respectively, indicating good and excellent performance (Figure 2). Simultaneously, the ER value informs us about the presence of activity from a certain model. Our ROC curve showed good results, with an AUC of 1.0, while ER values of 11.4 to 13.1 showed excellent outcomes with a value greater than 0.9. Analysis of the ROC curve found a total of 36 true positives (TF) and 3 false positives (FP) from the 472 compounds (Figure 2). The calculated GH and EF score has also been mentioned in Table S2.

### 2.3. Dataset Identification for Pharmacophore Screening

Dataset generation is essential for identifying lead compounds. The ZINC is the most commercially available database, containing 230 million purchasable compounds and 1.37 million compounds (including natural and synthetic compounds) ready-to-dock for achieving binding affinity and providing the 3D structures for clinical development [28]. We utilized the natural product database and the natural derivatives database, which contain millions of currently marketed drugs, natural products, and semisynthetic medical products. The database was initially filtered based on molecular weight <500 kDa and retrieved for the further screening process.

### 2.4. Pharmacophore Model-Based Virtual Screening

The validated pharmacophore model obtained from the LigandScout 4.4.8 advanced tools was used to screen the ZINC natural compounds library. Initially, the validated model was submitted in the ZINCPharmer pharmacophore search tools for screening the purchasable natural subset of the ZINC database. During the screening process, the molecular weight limit set was 500 kDA, and the RMSD was ≤1. One hydrophobic feature with the inclusion–exclusion volume was omitted before screening the ZINC natural compound library. The pharmacophore model-based virtual screening process identified 136 hits with fit scores ranging between 85.2 to 96.7 that were saved for further screening.

### 2.5. Docking-Based Virtual Screening

#### 2.5.1. Binding-Site Detection and Ligand-Protein Interaction Prediction

**Figure 3:**
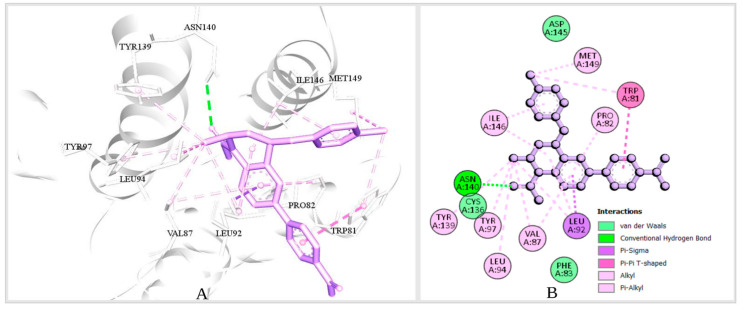
Binding sites of the protein. (**A**) The 3D representation of the binding-site residues of the protein retrieved from the PDB ID: 4BJX, and (**B**) the 2D representation of the binding-site residues of the protein-ligand interaction.

The desired Brd4 protein (PDB ID: 4BJX) had two associated ligands and separate attachment sites for interacting with the target ligand. The bond formation with the various amino acid (AA) residues of the protein was demonstrated by analyzing the protein-ligand complex structure using Discovery Studio software and a CASTp server (Figure 3A). The numbers of active sites interacting with the ligand are shown (Figure 3B), and the number of active sites determined by CASTp is provided in Figure S1A. The program was used to count the number of hydrophobic and hydrogen-bond-acceptor contacts between amino acids of the protein. The hydrogen-bond-acceptor contacts are represented by red arrows (Figure S1C). The Ramachandran plot was used to determine the beta-sheet, right-handed helix, and left-handed helix in the protein structure (Figure S1B).

#### 2.5.2. Molecular Docking

Docking is a technique used in drug development to assess the binding affinity of a protein and its ligand [29]. The method aids in predicting the behavior of proteins (enzymes) and their interactions with small molecules (ligands) to the binding sites of target proteins. Therefore, this study used a molecular docking simulation to determine the ligand-receptor binding process. The 136 hits screened through the structure-based virtual screen process were docked to the desired protein. Initially, the protein was prepared using the Biovia Discovery Studio tools by adding a force field, namely CHARMm. Later the binding site residues identified from the complex structure analysis process were used to make a receptor grid, where the grid dimension was X = 30.1404, Y = 25.00, and Z = 25.4020. The hits were docked with the intended protein using AutoDock Vina, and the four compounds with the highest binding energy (ZINC4104882, ZINC2509501, ZINC2566088, and ZINC1615112) were chosen for further analysis (Table 1). The four compounds chosen in the study were also validated through the re-docking process (Table S3). For docking validation, the same grid box position was used, and the docking was visualized, as shown in Figure S2.

#### 2.5.3. Analysis of Protein–Ligand Interaction

**Figure 4:**
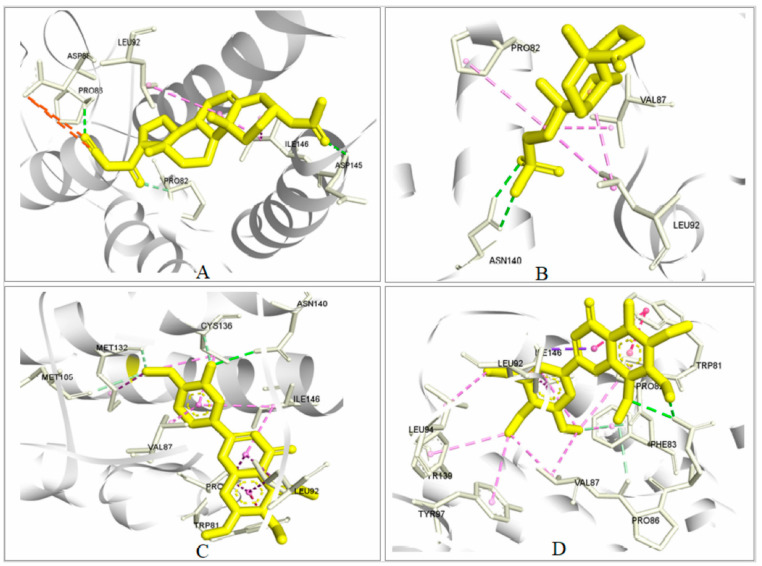
Three-dimensional interactions of the selected ligands with the protein complex. In our interactions, (**A**) ZINC4104882, (**B**) ZINC2509501, (**C**) ZINC2566088 and (**D**) ZINC1615112, and all showed better contact with the Brd4 protein. All four compounds were selected depending on the docking scores. The key amino acids in our interaction were LEU92, PRO82, GLN85, ASN140, and VAL87, and they were predominant in all the compound interactions.

**Figure 5:**
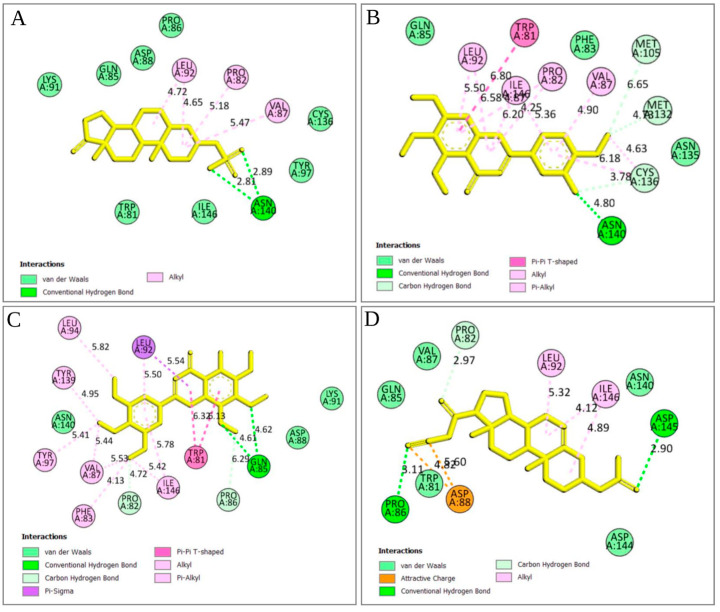
Two-dimensional (2D) interaction between four selected ligands with the Brd4 protein. In the ligand–protein interaction, (**A**) ZINC4104882, (**B**) ZINC2509501, (**C**) ZINC2566088, and (**D**) ZINC1615112 show amino acids binding with the BET family protein Brd4. Colors indicated the different types of bonds along with the distance, except van der Waals forces.

The strength of the ligand’s binding to the target protein was determined by analyzing the protein–ligand interaction. In the experiment, it was found that the stronger the binding affinity, the greater the interaction with the amino acids with the target protein. A total of four bonds were present in several amino acids (Figure 4 and Figure 5). Among them, there were two attractive charges at ASP88, one carbon–hydrogen bond (PR082), two conventional hydrogen bonds (GLN85 and ASP145), and two alkyl bonds (LEU92 and ILE146) in ZINC4104882 (binding affinity, −8.0 kcal/mol). ZINC2509501 formed three bonds with various amino acid positions in the Brd4 protein, including one conventional hydrogen bond (ASN140), three alkyl bonds (LEU92 and PRO82), and one unfavorable acceptor–acceptor bond (ASN140). For ZINC2566088 (binding affinity, −7.6 kcal/mol), most were pi–alkyl-type bonds (Figure 4 and Figure 5) with PRO82, LEU92, ILE146, PRO82, VAL87, CYS136, and ILE146. There were four carbon–hydrogen bonds (CYS136, MET105, and MET132), one pi–pi T-shaped bond (TRP81), and two alkyl bonds (MET132 and CYS136).

For ZINC1615112 (binding affinity, −7.2 kcal/mol), there were five pi–alkyl bonds (PRO82, VAL87, ILE146, LEU94, and VAL87), two carbon–hydrogen bonds (PRO86 and PRO82), two pi–pi T-shaped bonds (TRP81), and five pi–alkyl bonds (PHE83, THR97, THR139, LEU92, and ILE146).

#### 2.5.4. Pharmacophore Features Analysis

**Figure 6:**
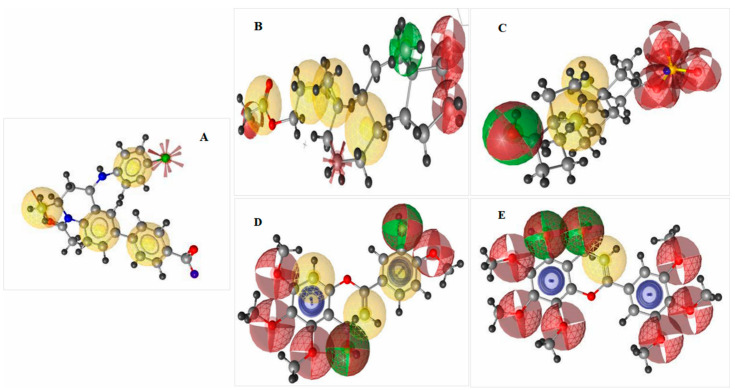
Pharmacophore feature analysis of four selected compounds with the target BRD4 protein. Herein, the figure representing (**A**) Query pharmacophore features and features generated from the attached ligand (ZINC95504909) with the selected protein Brd4 (PDB: 4BJX). The crystal structure generated four hydrophobic bonds (yellow), one negative ionizable group (red star), one H-bond acceptor (red), and one hydrophobic feature that shifted to a negative ionizable feature were detected. (**B**) ZINC2509501, (**C**) ZINC2566088, (**D**) ZINC1615112, and (**E**) ZINC4104882, the number of features was better than that for control ligand ZINC95504909 (**A**). Blue color indicated the presence of an aromatic ring among the selected ligand.

Prior to the development of medicine, lead development and screening were important aspects of the pharmaceutical company [14]. For the virtual scanning of the database library, many methods for identifying pharmacophore features are required. To optimize a biologically active compound, compounds with similar properties should be identified. Using the rule of five, we analyzed the drug-likeness and non-drug properties of the top four higher-binding-energy compounds, ZINC4104882, ZINC2509501, ZINC2566088, and ZINC1615112. The pharmacophore features generated from the selected compounds had better or similar properties with respect to the attached antagonist (ZINC95504909). Therefore, the selected compounds should have shown better efficacy against the Brd4 protein and, subsequently, the MYCN gene. The features obtained for the four selected compounds are shown in Figure 6.

### 2.6. MM/GBSA Analysis

The MM-GBSA techniques were employed to determine the binding free energy of the selected four compounds in complex with the targeted protein. The desired ligand confirmation obtained through the docking process was used to estimate the binding free energy [22,30]. The selected four compounds ZINC2509501, ZINC2566088, ZINC1615112, and ZINC4104882 in complex with the targeted protein show ∆G binding (∆GB) energies value −19.91 kcal/mol, −16.69 kcal/mol, −17.98 kcal/mol, −15.34 kcal/mol (Table 2). Additionally, analysis of the physical and chemical properties found an invaluable achievement of ∆G Bind Covalent (∆GBCo), ∆G Bind Hbond (∆GBH), ∆G Bind Lipo (∆GBL), ∆G Bind Packing (∆GBP), ∆G Bind Solv GB (∆GBS), and ∆G Bind vdW(∆GBV) energies for the selected four compounds listed in Table 2. Therefore, it can be said that the compounds would be able to make a prolog binding affinity with the desired Brd4 protein.

### 2.7. Pharmacokinetics and Toxicity Properties Analysis

#### 2.7.1. Pharmacokinetic (ADME) Evaluation

Absorption, distribution, metabolism, and excretion (ADME), combinedly known as the pharmacokinetics properties of a lead compound, play an important role in the likelihood of success of a drug. These parameters determine the kinetic behaviors of a drug inside the body from administration to excretion through sweating, urine, or feces [14]. The volume of the drug distributed to the tissue and target site is important for its bioavailability and for reducing the side effects and toxic effects. The determination of the efficacy and toxic effect of a drug before designing it for a clinical trial can help in evaluating the possible unwanted effects in human or animal models. Thus, the evaluation of the drug candidate by using the online server Swiss ADME may help to reduce the cost by minimizing drug rejection. We evaluated the ADME properties, such as the lipophilicity, water-solubility, drug-likeness, and medicinal chemistry of our four selected compounds. The ADME properties found for all of the selected compounds in the optimized range and listed in Table 3.

#### 2.7.2. Toxicity Determination

Toxicity analysis is a popular way to select an appropriate drug candidate through computer-based drug design due to its accuracy, rapidity, and accessibility for synthetic and natural compounds [12]. The TEST tool and ProTox-II are free tools that can be used to determine the toxicity of compounds. The cytotoxicity, mutagenicity, carcinogenicity, hepatotoxicity, and LD_50_ (mg/kg) were calculated using the tools listed in Table 4. According to ProTox II, ZINC2509501 belongs to class 6, meaning nontoxic. On the other hand, ZINC4104882, ZINC2566088, and ZINC1615112 belong to class 5, meaning they may be toxic if 5000 mg/kg is swallowed.

### 2.8. Protein–Ligand Interaction Analysis through Dynamic Simulation

Molecular dynamics simulations help atomic-scale investigations into the movement of atoms and molecules. It also provides the number of bond types and their interaction with the respective amino acids within a specific time [15]. The complex structure file obtained from the molecular docking was analyzed using the Schrödinger Desmond simulation tool.

#### 2.8.1. RMSD Value Analysis

In the dynamic simulation, the number of displaced atoms was generated concerning the time-generated RMSD (root mean square deviation) value. First, the protein Cα, backbone, side chains, and heavy atoms were determined, and protein-fitting ligands were also measured at the observed duration (100 ns).

##### Analysis of Protein RMSD

**Figure 7:**
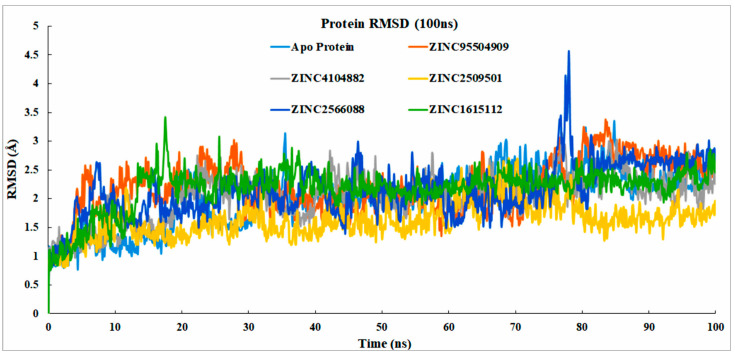
RMSD values of the protein were determined from the Cα of the protein–ligand docked complex. Apo-Protein (light blue), ZINC95504909 (orange), ZINC4104882 (gray), ZINC2509501 (gold), ZINC2566088 (blue), and ZINC1615112 (green).

Most of the compounds were stable based on the reference protein variability (1–3 Å) and their interactions with the ligand; values above 3 Å indicate large conformational changes in the protein, implying that the system is unstable. Analysis of the selected proteins (ZINC2509501, ZINC2566088, ZINC1615112, and ZINC4104882) and control ligand (ZINC95504909) showed that most were stable, although the ZINC2566088 compound had some fluctuation at 80 ns (4.59 Å). The other compounds were more stable (Figure 7) than the control compound (ZINC95504909).

##### Analysis of Ligand RMSD

**Figure 8:**
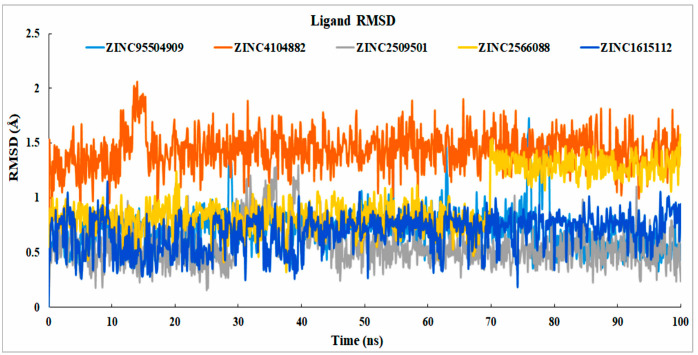
RMSD value determined from the protein-fitting ligand interaction. Different colors of the selected compounds, i.e., ZINC4104882 (orange), ZINC2509501 (gray), ZINC2566088 (gold), ZINC1615112 (blue), and control ligand ZINC95504909 (light blue) indicated the numbers of ligands and their expression with respect to the 100 ns simulation time.

The data gathered from the interactions between proteins and ligands showed that all the ligand interactions were stable. For the compounds, ZINC2566088 was unstable at 70 ns and stable from the beginning of 72 ns, but the compound did not exceed the RMSD by more than 2 Å. The selected control ligand (ZINC95504909) showed instability with respect to the protein at 30, 54.2, and 62.9 ns, and from 75.9 to 80.4 ns, it showed maximum fluctuations and, again, reached an equilibrium state. The natural compound ZINC2509501 was unstable from 25 to 39.5 ns; after that, it reached a stable state (Figure 8. From 0 to 35 ns, compound ZINC2509501 showed no fluctuations, but from 35 to 40 ns showed instability and again came to the stable expression. The stability of the ligand with respect to the protein and its binding pocket was determined by ligand RMSD analysis.

#### 2.8.2. RMSF Analysis

**Figure 9:**
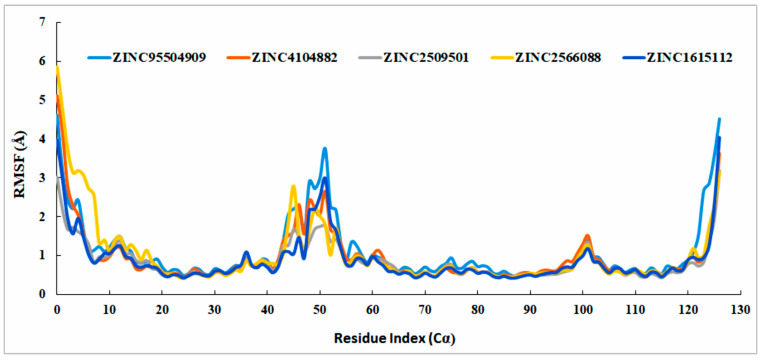
RMSF values of the all-natural compounds were determined from the protein Cα atoms. Several colors indicated our natural compounds i.e., ZINC4104882 (orange), ZINC2509501 (gray), ZINC2566088 (gold), ZINC1615112 (blue), and control ligand ZINC95504909 (light blue).

The root mean square fluctuation (RMSF) was used to evaluate the conformational changes between the compound and the selected ligands. All the compounds had minimal RMSF values, but most showed low instability at position 50 (LEU92). The maximum RMSF in the N-terminal was observed at a maximum of 4.52 Å. All the compounds had lower RMSF values, increasing the possibility of discovering new compounds targeting the Brd4 protein (Figure 9).

#### 2.8.3. Protein–Ligand Interaction Analysis

**Figure 10:**
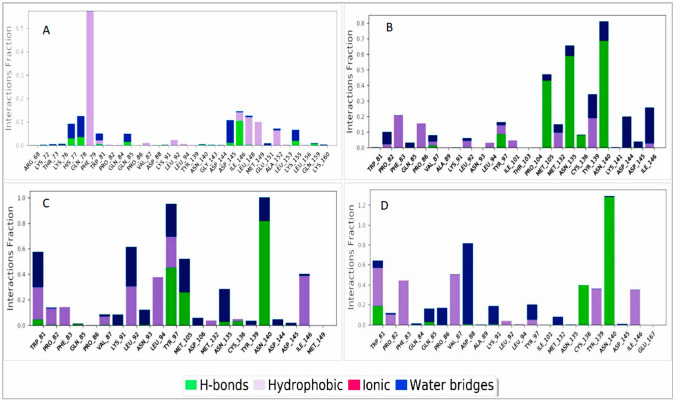
Protein–ligand interactions for the selected compounds: (**A**) ZINC4104882, (**B**) ZINC2509501, (**C**) ZINC2566088, and (**D**) ZINC1615112.

Several bonds were involved in the protein–ligand interactions, such as hydrogen bonds, hydrophobic interactions, and water bridges. These bonds are important when considering a compound as a drug molecule. In our experiment, all the compounds showed tangible contact with the targeted protein (Brd4) molecule. In ZINC2509501, the amino acids ALA89 and THR103 did not show contact with the protein (Figure 10B).

**Figure 11:**
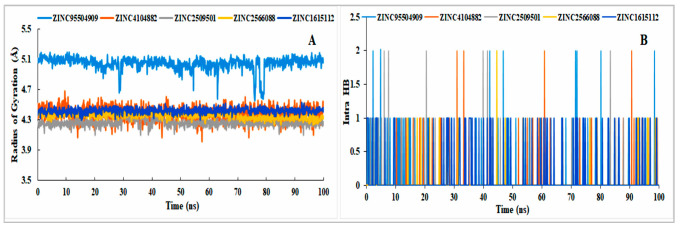
Analysis of the radius of gyration (**A**) and intra-molecular hydrogen bonds (**B**) among the selected ligands and control ligand (ZINC95504909). Different colors mentioned our natural compounds i.e., ZINC4104882 (orange), ZINC2509501 (gray), ZINC2566088 (gold), ZINC1615112 (blue), and control ligand ZINC95504909 (light blue).

All the selected ligands were compressed during the simulation time except the control ligand ZINC95504909, where the structural conformation changed highly (Figure 11A). The number of intra-molecular hydrogen bonds was higher in ligand ZINC1615112 and increased the binding properties to the protein (Figure 11B).

## 3. Discussion

Every year, more people die from cancer than other chronic diseases, such as HIV, malaria, and tuberculosis combined. In 2020, the death rate was close to 55%, compared to nearly 25% in 1960 [31]. Several genes are typically involved in carcinoma development through the deregulation of cellular growth, nucleic acid repair, chromosome instability, cell interaction and communication, angiogenesis, and changes in the apoptotic pathway [32,33].

Several factors are responsible for the delayed development of cancer among neuroblastoma patients, such as early diagnosis and molecular and genetic factors. Neuroblastoma has also been observed among children, with a mortality rate of 13% [34,35]. Nowadays, BET proteins are used as therapeutic targets because of research linking pharmacologic suppression to patterning in Brd4 localization on chromatin and/or modifications in overall genetic expression [36,37]. Brd4 is a crucial part of many illness-associated gene regulation networks. This could explain why BET proteins have recently become experimental targets in a remarkable variety of disease models, including cancer [18,37]. Neuroblastoma inhibition by targeting BET protein overexpression is a novel research finding, as previous studies revealed that several scientists tried to find a BET protein inhibitor using a combination of two drugs, some of which used recombinant protein inhibitors using a combination of two drugs with some of them using recombinant protein [38,39]. Current neuroblastoma treatment includes surgery, chemotherapy, and radiotherapy. The treatments improve the condition but are not patient-friendly due to their side effects. The MYCN oncogene has great potential to induce the development of more aggressive neuroblastomas. Most neuroblastoma cases initially respond to treatment, but the treatment ultimately fails due to drug resistance [40]. BET inhibitors are becoming resistant in cancer therapy due to increased Wnt signaling catenin-mediated MYC in AML and activation of the RAS pathway in lymphoma [1]. The proto-oncogenes MYC was transcriptionally suppressed by Brd4-NUT oncoprotein, released by the presence of BET inhibitors [41]. BET inhibitors’ anti-cancer efficacy can occasionally be linked to their effects on MYC transcription. Still, in other cases, these effects are controlled by a distinct group of cancer-related genes [42].

The study indicated the possibility of developing a new class of compounds that would be more suitable for cancer patients due to the lower side effects but needs further experimental and animal studies. We first selected a suitable protein candidate from the protein data bank, and the currently available antagonists were identified from a literature search. The number of fit compounds with pharmacophore features similar to those of the attached ligand was retrieved from the online database [22]. The obtained compounds were validated by downloading all the structures of the 36 antagonists with the specific numbers of decoy sets from the Ligand Scout 4.4 software [43,44]. The ROC curve was satisfactory, and the validated models were further used to generate fit compounds. All the compounds were transferred to the PyRx software to obtain the binding affinity for the fit compounds by molecular docking [30]. The top four compounds with higher binding affinity and lower side effects, as determined by the software, were selected for further experiments. All the selected compounds were evaluated for their pharmacokinetic properties, such as absorption, distribution, metabolism, and excretion [22]. All the compounds had better absorption capability and drug-likeness properties, as there was no violation of the rule of five; they also had better bioavailability scores. The compounds with good ADME profiles were used for further toxicity analysis [45].

The molecular dynamic simulation provided us with data regarding the stability of the binding between proteins and ligands for 100 ns. The developed trajectory file was further studied to find the RMSD and RMSF values of both the proteins and ligands as well as the protein structure predictions, also the numbers of hydrophobic, hydrophilic, H-bond, ionic, and water bridges [12]. This identification of potential lead natural compounds would be able to inhibit the Brd4 protein in neuroblastoma treatment by silencing the expression of MYCN. This research focused on identifying potentially natural compounds that would be more patient-friendly, due to having fewer toxic effects and side effects, for treating MYCN overexpressing neuroblastoma patients by targeting the Brd4 protein.

## 4. Materials and Methods

### 4.1. Generation of the Pharmacophore Model

#### 4.1.1. Structure-Based Pharmacophore Modeling

The crystallographic structure of the Brd4 protein was obtained from the PDB database (PDB ID: 4BJX) and filtered depending on the protein resolution (1 to 2.0 Å). The structure-based preparation protein was selected by the attachment of ligands. Meanwhile, the currently available antagonists were identified by a literature search and from the ChEMBL database (https://www.ebi.ac.uk/chembl/; accessd on 10 May 2022). In total, 36 antagonists were identified (Table S1) from online databases and rigorous literature searches. The ligands were screened and examined to determine their toxicity (through the ProTox-II server; (https://tox-new.charite.de/protox_II/; accessd on 9 May 2022) and inhibitory activity in neuroblastoma. All the antagonists underwent docking, and the best one was selected for structure-based drug design. The selected proteins (PDB ID: 4BJX) were analyzed using the LigandScout 4.4.8 Advance software for the number of hydrogen-bond donors or acceptors, hydrophilicity, lipophilic characteristics, and ionizable charges. A total of five hydrophobic and five hydrogen-bond acceptors were determined. For better results, we followed the Lipinski rule of five and added one positive feature from the protein for better model generation.

#### 4.1.2. Model Validation

Validation was essential when evaluating the quality of our model through protein–ligand interactions; several known active inhibitors (Table S1) were used to distinguish between the selected active and inactive compounds. Compounds with larger molecular weights and higher IC_50_ values were not selected [18,25]. The identified active compounds (Table S1) were evaluated using the DUD-E decoy set (obtained from the DUD-E decoys database) for better measurement between the active and inactive compounds. The selected DUD-E decoy and our compounds were converted to an “idb” file using LigandScout before preparing an ROC curve. The quality of the curve was evaluated based on the true and false positives, enrichment factor, and GH score. The accuracy of the model was examined considering important parameters such as the active hits (AH), decoy compounds (DC), early enrichment factor (EF), overall compounds in the dataset (D), the total number of hits retrieved (TH), and goodness of hit score (GH).

#### 4.1.3. Dataset Preparation and Virtual Screening

Identifying structurally similar novel and active molecules is important for structure-based drug discovery based on the prepared pharmacophore model. Freely accessible ZINC databases containing 230 million compounds were screened for the further identification of structurally similar compounds. The 2D and 3D structures and physical and chemical properties can therein be obtained for a compound [46,47]. The compounds most similar to our selected ligand features were selected. Several libraries were available in ZINC Pharmer (http://zincpharmer.csb.pitt.edu/; accessd on 11 May 2022) for dataset generation, and we used the ZINC natural compound library for searching compounds by matching the maximum features. The pharmacophore features of the selected compound were analyzed and prepared for screening depending on adding one feature and omitting exclusion volumes. A total of 136 hits were retrieved (Supplementary file renamed Hits), with the maximum query features considered for further validation. The compounds were further analyzed by ligand scout 4.4 software to generate the pharmacophore fit score.

### 4.2. Molecular-Docking-Based Virtual Screening

#### 4.2.1. Protein and Ligand Preparation

The macromolecular structure of the protein obtained from the PDB was prepared for molecular docking. The protein’s crystal structure (PDB ID: 4BJX) attached to the 73B ligand was used as a positive control, and the attached ligand was separated using the Discovery Studio Visualizer (version 16.1.0). Adding any necessary bond and deletion of the water molecules was not part of the structural refinement process. The desired protein structure was obtained and analyzed, determining the free R value (0.185), resolution (1.59 A), and observed R value (0.158). A few bonds were missing in the currently selected protein, and we used Discovery Studio to attach a new bond by adding the force field (CHARMm). All the ligands obtained through screening the ZINC database were prepared and optimized using the OPLS_2005 force field Schrodinger Suit. The ionization pH range was set to 7.1 ± 2.

#### 4.2.2. Grid Preparation and Active-Site Identification

The prepared structure of the selected protein was analyzed using UniProtKB and PrankWeb (https://prankweb.cz/; accessd on 11 May 2022) to identify the active site of the protein. Furthermore, CASTp (CASTp 3.0: Computed Atlas of Surface Topography of proteins (uic.edu)) was used to identify the number of active pockets (Figure S1). The binding affinity of the protein and ligand depends on the presence of hydrogen bonds, lipophilic or hydrophilic interactions, and ionizable charges. Additionally, we used the PrankWeb (http://prankweb.cz/; accessd on 11 May 2022) database for active-site identification. Using the PyRx tool, the receptor grid was prepared through selecting the active site of the protein.

#### 4.2.3. Binding Affinity Determination by Docking

Identified hits of the compound’s 2D structure were downloaded from the NCBI databank and the PubChem compounds. All the compounds were transferred to the PyRx software, and docking was carried out using the AutoDock Vina tools. PyRx is a popular tool in drug design for selecting a drug active against different animal diseases to identify potential drug candidates. Based on the binding affinity and RMSD value, the compounds were further transferred to the BIOVA Discovery Studio Visualizer Tool 16.1.0 for analyzing the interaction [22,44].

### 4.3. MM/GBSA Data Calculation

The molecular mechanics generalized Born surface area (MM/GBSA) is a popular method usually used to calculate the free binding energy of ligands [48]. We used the Prime MM/GBSA package from Schrödinger (released in March 2020) to evaluate the free binding energy.

### 4.4. Toxicity Class Prediction and Evaluation for Drug Candidate

#### 4.4.1. Pharmacokinetic and Pharmacodynamic Evaluation

The most important parameters to consider for treating disease are a drug’s absorption, distribution, metabolism, and excretion after administration inside the body by any of the available routes (oral, nasal, intravenous, subcutaneous, and rectal) [49,50]. The metabolism of a drug by different enzymes and microbiota may affect its release and cause it to produce toxic effects or change its efficacy [51]. Computer-aided drug design has emerged as a new pathway for choosing the best candidate for drug development before proceeding to the clinical trial phase [22]. A drug’s bioavailability is affected by gender, age, disease condition, lipophilicity, hydrophobicity, microbiota, body enzymes, and administration pathway [52]. We used the freely accessible database Swiss ADME (http://www.swissadme.ch/index.php; accessd on 13 May 2022) to identify the solubility pattern, enzymatic degradation, and absorption for each selected compound.

#### 4.4.2. Toxicity Analysis

Measuring a drug’s toxicity is important in establishing a drug candidate for drug discovery and development. Computer-based drug design has made it easy to determine toxicity profiles (hepatic failure, carcinogenicity, immune response, and membrane potential pathway) both quantitatively and qualitatively [25,53]. Toxicity measurement tools (Toxicity Estimation Software Tool, TEST version 4.2.1) are usually used to determine the toxic effects of chemicals based on their molecular structures. Our toxicity study (Table 4) determined the fathead minnow LC_50_ (96 h), 48-h daphnia magna LC_50_, developmental toxicity, oral rat LD_50_, bioaccumulation factor, and water solubility (at 25 °C). The hepatotoxicity, carcinogenicity, mutagenicity, immunogenic response, and several toxicological pathways were determined using the ProTox-II server (https://tox-new.charite.de/protox_II/; accessd on 13 May 2022).

### 4.5. Molecular Dynamic Simulation

#### 4.5.1. Preparation of the Protein and Ligand for Simulation

To justify our ligand’s binding to the protein after it was obtained from the docking studies, we performed a 100 ns dynamic simulation. It was necessary to determine the complex’s stability and predict every atom-binding movement of the ligand and protein molecules over a specific period. Our dynamic simulation was processed using the commercial software Schrödinger Release 2020-3 (Academic version) with the Linux command line. The protein–ligand interaction was first solved with the water model, and an orthorhombic box shape boundary was obtained. The complex atom buffer box calculation method was used by combining Na^+^ and Cl^−^ to create a 0.15 M sodium chloride solution. The simulation was carried out with an ensemble temperature of 300 K at a pressure of 1.01325 bar, with a recording at 50 ps. The simulation was run using the OPLS-2005 force field.

#### 4.5.2. Trajectory File Analysis

All the trajectory files from the simulation were analyzed using the Simulation Interaction Diagram (SID) of the Desmond module in the Schrödinger package. The simulation trajectory file provided information regarding the stability of the protein–ligand interaction complex based on the RMSD and RMSF values and ligand–protein complex. The radius of gyration among the selected ligand was evaluated to measure the extendedness of the ligand, and the intra-molecular hydrogen bond was determined.

## 5. Conclusions

The study discovered four natural compounds ZINC2509501, ZINC2566088, ZINC1615112, and ZINC4104882—which can attach to the Brd4 protein and have the possibility to block the activity of this protein overexpression and subsequently suppress the MYCN overexpression expression. All the substances had greater protein-binding affinities, ranging between −7.6 to −8 kcal/mol. The selected compounds show substantially improved pharmacophore features compared to the compound (PDB:73B) attached to the Brd4 (PDB ID: 4BJX) protein. The ADME profile of the selected four natural compounds has a good value that maintains all the rules of the Lipinski rule of five. Additionally, a computer-based toxicity study revealed that all the compounds were less harmful than the currently available medications for treating neuroblastoma patients. The MD simulations revealed the ligand’s stability in its interaction with the protein complex for 100 ns. The computational study suggested four compounds that have the potential to combat neuroblastoma and may aid in reducing neuroblastoma-related deaths in children.

## Figures and Tables

**Table 1:** List of the top four compounds and their chemical name, molecular formula, and binding affinity (kcal/mol) of the selected four compounds. The compounds were selected based on the binding affinity score obtained from the molecular docking experiment.

ZINC ID	Compound Name	Molecular Formula	Binding Affinity (kcal/mol)
ZINC4104882	[17-(2-diazoacetyl)-10,13-dimethyl-2,3,4,7,8,9,11,12,14,15,16,17-dodecahydro-1H-cyclopenta[a]phenanthren-3-yl] acetate	C_23_H_32_N_2_O_3_	−8.0
ZINC2509501	[(3S,8R,9S,10S,13S,14S)-10,13-Dimethyl-17-oxo-1,2,3,4,5,6,7,8,9,11,12,14,15,16-tetradecahydrocyclopenta[a]phenanthren-3-yl] hydrogen sulfate	C_19_H_30_O_5_S	−7.9
ZINC2566088	3′-Hydroxy-5,6,7,4′-tetramethoxyflavone	C_19_H_18_O_7_	−7.6
ZINC1615112	Gardenin A	C_21_H_22_O_9_	−7.2

**Table 2:** A list of different energy components and net MM/GBSA binding free energy (kcal/mol) and standard deviation values generated from the docking poses of the selected four compounds, including the control ligand (ZINC95504909).

Complex Name	∆GB	∆GBC	∆GBCo	∆GBH	∆GBL	∆GBP	∆GBS	∆GBV
ZINC4104882	−15.34 ± 1.04	−14.85 ± 2.22	2.102 ± 0.96	−1.87 ± 0.76	−7.56 ± 2.72	−0.331 ± 0.11	12.028 ± 2.09	−22.755 ± 3.77
ZINC2509501	−19.91 ± 2.92	−15.95 ± 1.36	2.260 ± 0.55	−2.89 ± 0.52	−7.48 ± 2.39	−0.315 ± 0.20	14.85 ± 2.69	−23.67 ± 2.11
ZINC2566088	−16.69 ± 1.53	−14.75 ± 2.36	2.80 ± 0.104	−1.70 ± 0.81	−5.48 ± 2.52	−0.34 ± 0.12	15.82 ± 1.66	−26.33 ± 2.45
ZINC1615112	−17.98 ± 2.19	−15.33 ± 1.017	2.58 ± 0.407	−1.08 ± 0.64	−8.70 ± 1.68	−0.21 ± 0.11	13.93 ± 1.07	−18.26 ± 1.78
ZINC95504909	−14.45 ± 2.04	−12.51 ± 1.33	2.22 ± 0.98	−2.22 ± 0.98	−4.56 ± 2.12	−0.451 ± 0.13	11.028 ± 1.08	−20.566 ± 2.88

**Table 3:** Identification of different properties of our four selected compounds showing the different physical, chemical, pharmacokinetic, and drug-likeness properties.

Properties	Parameter	ZINC4104882	ZINC2509501	ZINC2566088	ZINC1615112
Physico-chemical properties	MW (g/mol)	384.51	370.50	358.34	418.39
	Heavy atoms	28	25	26	30
	Arom. heavy atoms	0	0	16	16
	Rotatable bonds	4	2	5	7
	H-bond acceptors	5	5	7	9
	H-bond donors	0	1	1	1
	Molar refractivity	108.36	96.29	95.91	108.89
Lipophilicity	Log P_o/w_	3.48	2.48	3.31	3.79
Water solubility	Log S (ESOL)	Moderate	Soluble	Moderate	Moderate
Pharmacokinetics	GI absorption	High	High	High	High
	CYP3A4 inhibitor	No	No	Yes	Yes
	BBB permeant	No	No	No	No
Drug likeness	Lipinski violation	Yes	Yes	Yes	Yes
	Bioavailability score	0.55	0.56	0.55	0.55
Medi. chemistry	Synth. accessibility	5.23	4.82	3.57	3.91

BBB: Blood Brain Barrier; GI: Gastro Intestinal; MW: Molecular Weight; Medi. chemistry: Medicinal chemistry; Synth. accessibility: Synthetic accessibility.

**Table 4:** Analysis of several aspects of toxicity (organ toxicity, toxicity class, Tox21-nuclear-receptor signaling pathways, Tox21-stress-response pathway, fathead minnow LC_50_ (96-h), developmental toxicity, water solubility, oral rat LD_50_, and bioaccumulation factor) of the four selected compounds.

Endpoint	Target	ZINC4104882	ZINC2509501	ZINC2566088	ZINC1615112
Organ toxicity	Hepatotoxicity	Inactive	Inactive	Inactive	Inactive
Toxicity endpoints	Carcinogenicity	Active	Inactive	Inactive	Inactive
	Immunotoxicity	Active	Inactive	Active	Active
	Mutagenicity	Inactive	Inactive	Inactive	Inactive
	Cytotoxicity	Active	Inactive	Inactive	Inactive
	LD_50_ (mg/kg)	5000	6000	4000	5000
	Toxicity class	5	6	5	5
Tox21-nuclear-receptor signaling pathways	Androgen receptor (AR)	Active	Inactive	Inactive	Inactive
	Aryl hydrocarbon receptor (AhR)	Inactive	Inactive	Active	Active
Tox21-stress-response pathway	Heat shock factor response element	Inactive	Inactive	Inactive	Inactive
	Mitochondrial membrane potential (MMP)	Inactive	Inactive	Active	Active
	Phosphoprotein (tumor suppressor) p53	Inactive	Inactive	Inactive	Inactive
Fathead minnow LC_50_ (96 h)	mg/L	N/A	4.99	0.53	0.55
48-h Daphnia magna LC_50_	mg/L	N/A	N/A	8.48	15.42
Developmental toxicity	value	0.96	1.19	0.84	0.76
Oral rat LD_50_	mg/kg	N/A	N/A	142.54	972.5
Bioaccumulation factor	Log10	N/A	N/A	1.76	1.94
Water solubility (25 °C)	mg/L	N/A	32.56	57.67	27.25

LC_50_: Lethal Concentration 50, LD_50_: Lethal Dose 50.

## Data Availability

Data can be provided based on the request to the corresponding author.

## Supplementary Materials

The following supporting information can be downloaded at: https://www.mdpi.com/article/10.3390/cimb44100329/s1, Figure S1. Structural description and the probable active site for the ligand molecule. The LigandScout software was used to determine the interaction with the ligand. Figure S2: Binding process validation of the docking results. Table S1: List of known antagonists for the target protein, with the PubChem CIDs, molecular weights, and IC_50_. The binding affinity of each antagonist is provided with the docking score. Table S2: Enrichment factor and goodness of hit score of the model are included in the validation of the pharmacophore model utilizing the GH scoring method. Table S3: The docking of all selected compounds is provided with the standard deviation.
