# Supplementary material for: Pharmacophore-Model-Based Virtual-Screening Approaches Identified Novel Natural Molecular Candidates for Treating Human Neuroblastoma
Source: Curr Issues Mol Biol. 2022 Oct 13;44(10):4838–58. doi: 10.3390/cimb44100329 (PMC9600652; doi:10.3390/cimb44100329)

Table S1: List of known antagonist against Brd4 protein with the Pub Chem CID, Molecular weight, IC_{50}. The binding affinity of each antagonist has been provided with the docking score.

PubChem CID	Molecular Formula	Chemical Structure	Molecular Weight	IC_{50} (uM)	Binding Affinity (kcal/mol)
145986197	$C_{23}H_{14}N_6O$	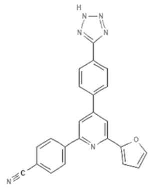	390.4	2	-6.8
145985926	$C_{22}H_{14}N_4O_2$	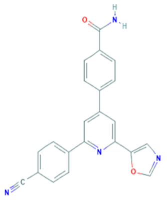	366.4	100	-7.0
145985805	$C_{25}H_{17}N_3O_2S$	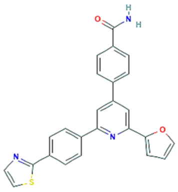	423.5	5	-7.2
145985420	$C_{25}H_{17}N_3O_3$	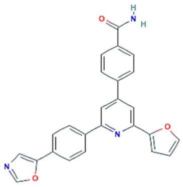	407.4	2.5	-7.3
145984862	$C_{25}H_{16}FN_3O$	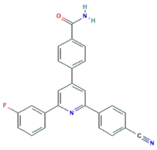	393.4	100	-7.1
**145984773**	$C_{22}H_{16}N_6O$	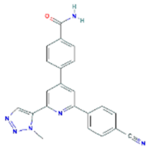	380.4	40	-7.0
145984250	$C_{23}H_{15}N_5O_2$	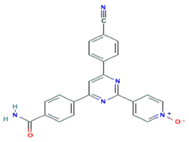	393.4	25	-6.8
145983842	$C_{21}H_{13}N_3OS_2$	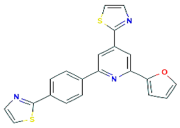	387.5	1.0	-7.1

145982841	$C_{24}H_{17}N_3O_4S$	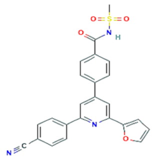	443.5	10	-6.7
127045497	$C_{20}H_{19}N_5O_2$	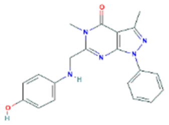	361.4	19.5	-6.8
119057309	$C_{26}H_{24}N_4O_2$	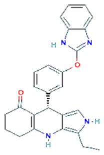	424.5	0.9	-7.1
118737586	$C_{25}H_{24}N_2O_7$	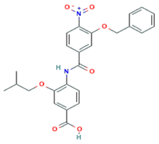	464.5	43	-6.7
118737578	$C_{29}H_{26}N_2O_7$	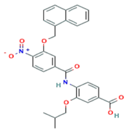	514.5	10.4	-6.9
118737557	$C_{21}H_{25}N_3O_7$	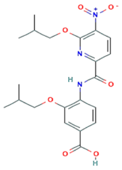	431.4	0.54	-6.9
118737556	$C_{20}H_{24}N_4O_7$	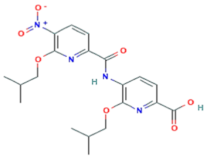	432.4	23.8	-7.2
118737551	$C_{26}H_{32}N_2O_8$	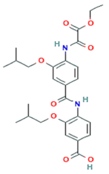	500.5	5.6	-6.5
118153396	$C_{23}H_{15}N_3O_2$	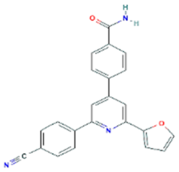	365.4	3	-7.2

88989242	$C_{18}H_{22}N_2OS_2$	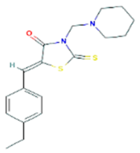	346.5		-6.4
71273226	$C_{35}H_{46}BrCl_2N_5O_9S_2$	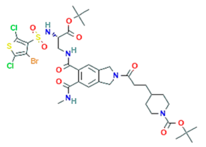	895.7	20	-7.0
70645391	$C_{26}H_{24}N_4O_2$	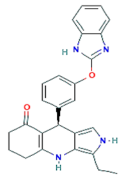	424.5	0.001	-7.3
54755136	$C_{22}H_{26}N_2O_7$	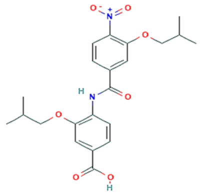	430.5	20.2	-6.5
52943159	$C_{36}H_{48}BrCl_2N_5O_8S_2$	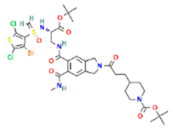	893.7	15	-7.3
46882744	$C_{21}H_{21}ClN_4O_5$	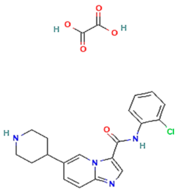	444.9	0.079	-3.8
9549289	$C_{18}H_{14}N_4O_2$	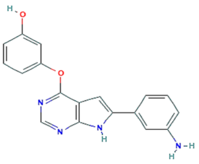	318.3	1	-7.3
6038254	$C_{16}H_{17}ClN_2OS_2$	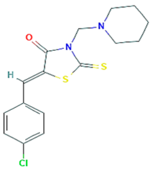	352.9	38	-6.1
52912222	$C_{25}H_{23}ClN_2O_3$	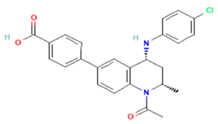	434.9	0.00025	-7.1

2836606	$C_{13}H_8N_4O_5$	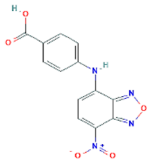	300.23	3	-7.5
2836600	$C_{18}H_{12}N_4O_3$	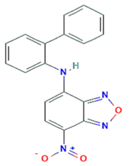	332.3	2.8	-7.5
2290404	$C_{15}H_{15}NOS_2$	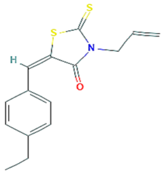	289.4	3.1	-5.9
1380234	$C_{10}H_6ClNOS_2$	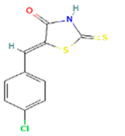	255.7	4.1	-5.5
1377581	$C_{13}H_{13}NOS_2$	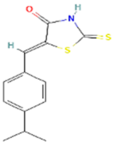	263.4	36	-6.6
1271003	$C_{12}H_{11}NOS_2$	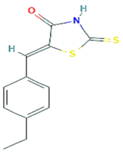	249.4	28.9	-5.8
445154	$C_{14}H_{12}O_3$	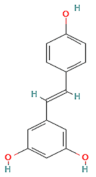	228.24	0.13	-7.0
208944	$C_{24}H_{29}NO_4S$	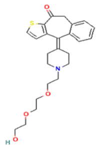	427.6	21	-6.3
5990469	$C_{19}H_{24}N_2OS_2$	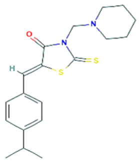	360.5	29	-6.1
44202126	$C_{41}H_{64}O_{13}$	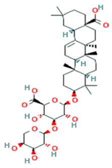	764.9	20.592	-6.9

Validation calculation: The total amount of GH and ER score has been calculated from the following equation one and two.

Table S2: Enrichment factor and goodness of hit score of the model are included in the validation of the pharmacophore model utilizing the GH scoring method.

Parameter	Symbol	Calculation	Model
Total compounds in database	D	–	472
Total actives in database	A	–	36
Decoy compounds	D_C	D-A	436
Total hits retrieved	T_H	–	39
Active hits retrieved	A_H	–	36
% yield of active hits	–	$(A_H/T_H)\times100$	92.307%
% ratio of active hits	–	$(A_H/A)\times100$	100%
Enrichment factor	EF	Eq. (1)	12.10
False negatives	F_N	$A - A_H$	0
False positives	F_P	$T_H - A_H$	3
Goodness of hit score	GH	Eq. (2)	0.936

Enrichment factor (EF) $= \frac{A_H \times D}{T_H \times A}$ (S1)

The goodness of hit Score (GH) $= [\{\frac{A_H \times (3A+T_H)}{4T_H \times A}\} \times \{1 - \frac{(T_H - A_H)}{(D-A)}\}]$ (S2)

where, AH is the active hits in the database, TH is the total number of hits retrieved, D is the total compounds in the database, A is the total number of actives in the database, (TH − AH) = FC is the number of false positives compounds, and (D − A) = DC is the decoy compounds of the database.

Table S3: The docking of the all selected compounds are provided with the standard deviation.

	ZINC95504909	ZINC4104882	ZINC2566088	ZINC2509501	ZINC1615112
	-7.1	-8.6	-7.7	-7.9	-7.2
	-7.1	-8	-7.6	-7.9	-7.2
	-7.1	-7.7	-7.8	-7.1	-6.9
Average	-7.1	-8.1	-7.7	-7.7	-7
STDEV	0	0.45825756	0.1	0.46188021	0.17320508

Figure S1. Structural description and the probable active site with the ligand molecule. The interaction with the ligand was determined by the ligand scout

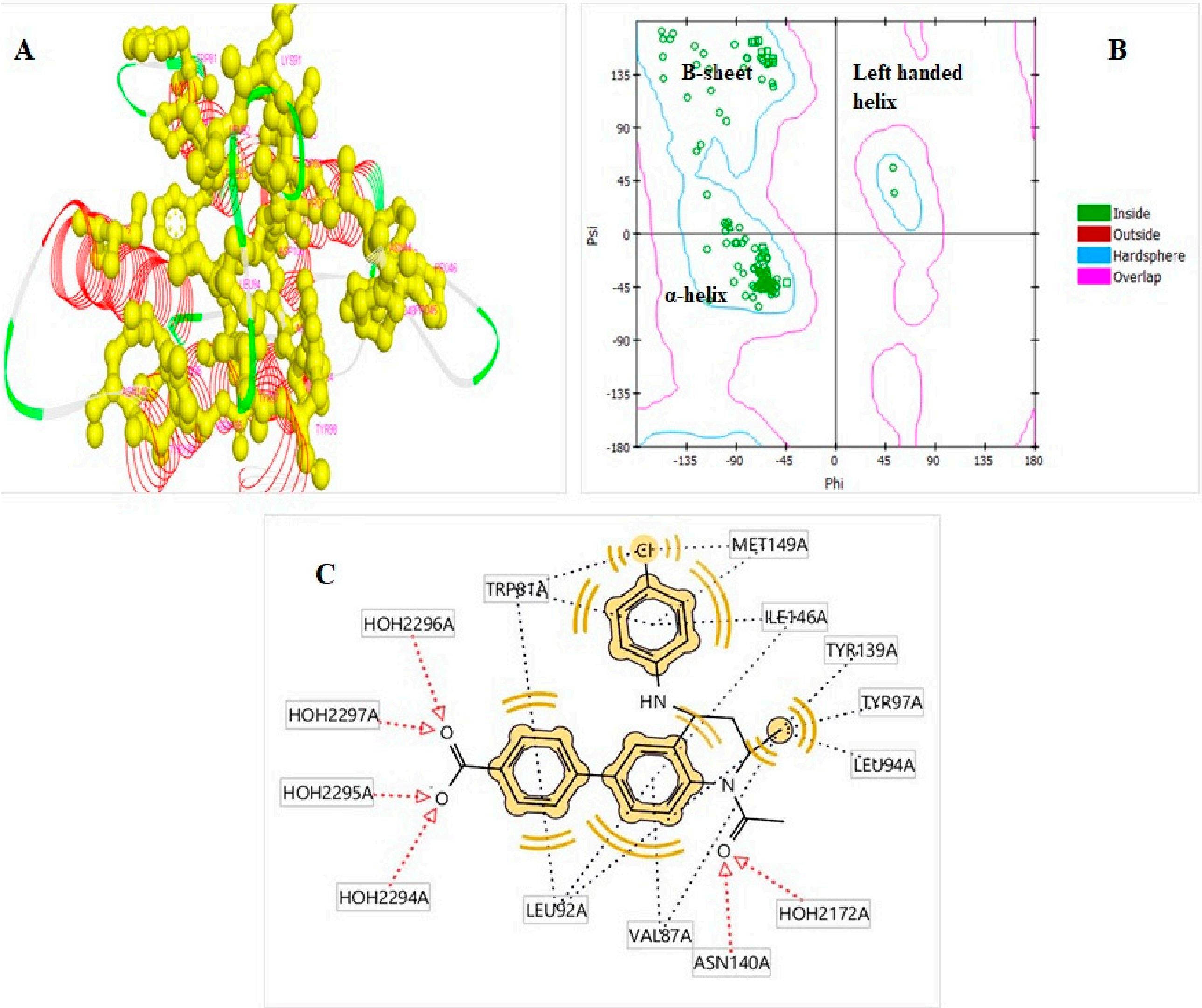

Figure S2: Binding process validation of the docking results.

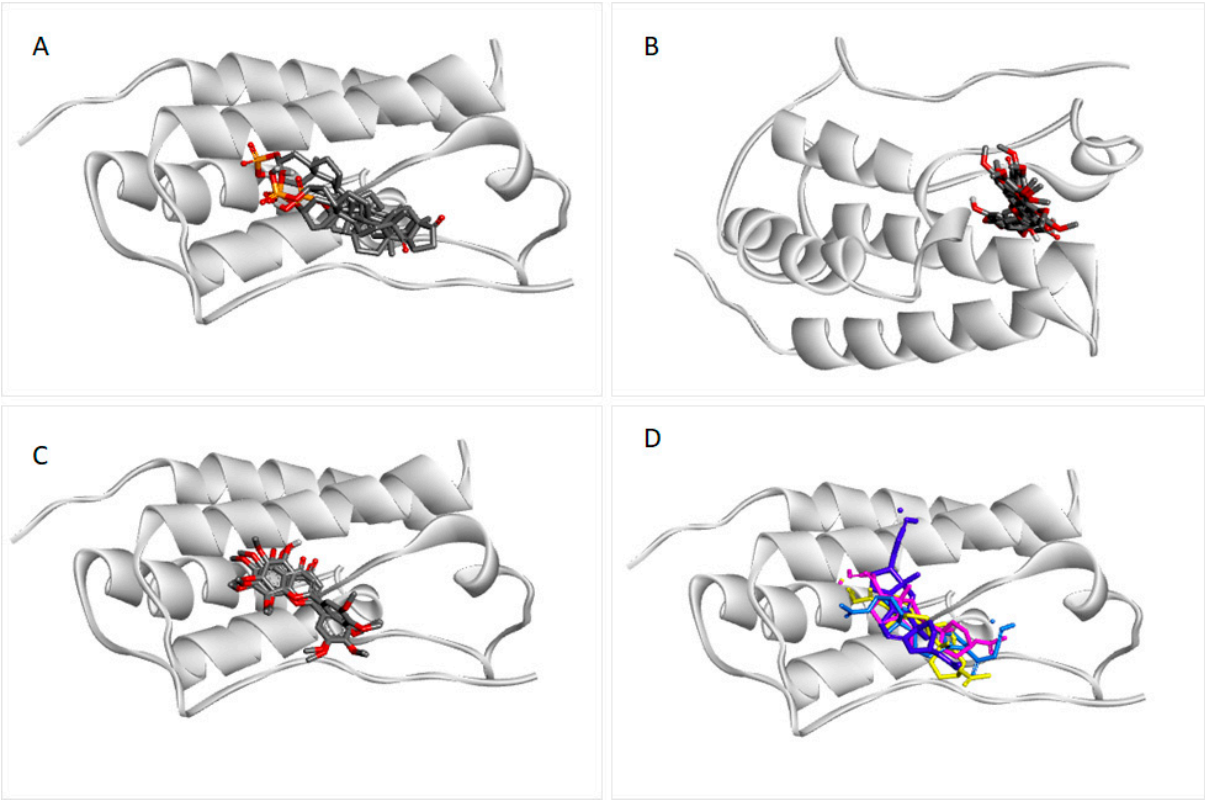

Supplement: Supplementary file 1 [file cimb-44-00329-s001.zip › Supplimentary Table and Figure.pdf]
